# Spt6 prevents transcription-coupled loss of posttranslationally modified histone H3

**DOI:** 10.1038/srep02186

**Published:** 2013-07-15

**Authors:** Hiroaki Kato, Kosuke Okazaki, Tetsushi Iida, Jun-ichi Nakayama, Yota Murakami, Takeshi Urano

**Affiliations:** 1 Department of Biochemistry, Shimane University School of Medicine, Izumo, Japan; 2 PRESTO, Japan Science and Technology Agency (JST), Saitama, Japan; 3 Division of Cytogenetics, National Institute of Genetics, Mishima, Japan; 4 Graduate School of Natural Sciences, Nagoya City University, Nagoya, Japan; 5 Laboratory for Chromatin Dynamics, RIKEN Center for Developmental Biology, Kobe, Japan; 6 Laboratory of Bioorganic Chemistry, Department of Chemistry, Faculty of Science, Hokkaido University, Sapporo, Japan

## Abstract

The tail of histone H3 is an ideal medium for storing epigenetic information because displacement of histone H3 is heavily restricted during transcription. To maintain the locus-specific modifications of histone H3, histone molecules should be retained locally at the original position through multiple rounds of transcription. Here, we found that fission yeast Spt6, a highly conserved RNA polymerase II-interacting histone H3–H4 chaperone, is essential for the maintenance of Lys-4 and Lys-9 methylation of histone H3 in euchromatin and heterochromatin, respectively. In euchromatin, loss of Lys-4 methylated histone H3 and deposition of newly synthesized Lys-56 acetylated histone H3 induced by Spt6 inactivation were coupled with transcription. While in heterochromatin, Spt6 prevents histone turnover and cryptic transcription in parallel with Clr3 histone deacetylase. We propose that Spt6 retains posttranslationally modified histone H3 during transcription to maintain epigenome integrity.

Posttranslational modifications of histone molecules epigenetically control the state of the corresponding genomic loci in *cis*^1^. Transcription by RNA polymerase II (Pol II) can cause dissociation of pre-existing histone molecules *in vitro*^2^,^3^ and *in vivo*^4^,^5^,^6^,^7^,^8^,^9^. Therefore, factors that oppose this dissociation by serving as molecular liaisons between histones and DNA should exist to ensure the integrity of the epigenome. Here, we show that Spt6 (Suppressor of Ty 6), a highly conserved histone H3–H4 chaperone that interacts with elongating Pol II^10^,^11^,^12^,^13^,^14^, is one such molecular liaison candidate. Spt6 has been shown to play important roles in nucleosome restoration in highly transcribed regions^11^, in genetic alterations at immunoglobulin loci^15^,^16^, and in methylation of Lys-4 and Lys-9 in histone H3^16^,^17^. However, the primary function of Spt6 in epigenetic regulation remains unclear.

We found that Spt6 prevents transcription-coupled nucleosome loss and loss of associated methylation of histone H3 at Lys-4 and Lys-9 in euchromatin and heterochromatin, respectively. The nucleosome loss caused by inactivation of Spt6 appears to be partially compensated for by Spt6-independent deposition of newly synthesized Lys-56 acetylated histone H3 molecules that do not carry locus-specific modifications. In other words, Spt6 prevents transcription-coupled histone turnover to maintain the posttranslational modifications of histone H3. We observed genetic interaction between Spt6 and Clr3 (cryptic loci regulator 3), a histone deacetylase that prevents histone turnover in heterochromatin^18^, indicating that Spt6 acts in parallel with Clr3^HDAC^ to prevent histone turnover and thereby maintain heterochromatin. These results strongly suggest that the primary function of Spt6 is to maintain epigenomic integrity by retaining posttranslationally modified histone H3 during transcription.

## Results

### Spt6 is required for the maintenance of Lys-9 dimethylated histone H3 in heterochromatin

**Figure 1:**
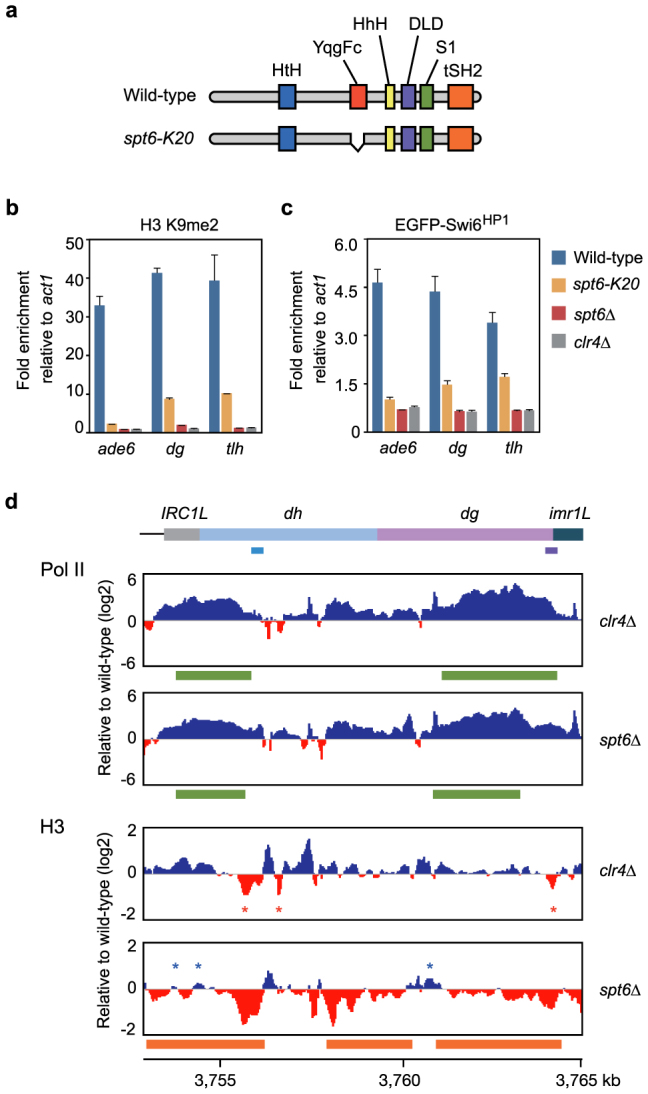
Spt6 is essential for the maintenance of Lys-9 dimethylated histone H3 in heterochromatin. (a) Schematic representation of the *spt6-K20* mutation. (b, c) ChIP-qPCR analysis of K9me2 (b) and EGFP-Swi6^HP1^ (c). Error bars, s.d. (d) ChIP-seq analysis of Pol II and histone H3 around the left side of centromere 1. Colored boxes at the top indicate the positions of pericentromeric repeat elements. The qPCR targets (*dh* in light blue and *dg* in purple) are shown. Positive (blue) and negative (red) values are filled with indicated colors. Red asterisks indicate the positions of nucleosome loss in *clr4Δ*^Suv39h^ cells. Blue asterisks indicate the positions where nucleosome loss does not occur in *spt6Δ* cells. Regions with a significant increase in Pol II occupancy (green) and significant decrease in histone H3 occupancy (orange) as detected by MACS^37^ are shown.

In a forward genetic screen for heterochromatin regulators that function in conjunction with Pol II^19^ in the fission yeast *Schizosaccharomyces pombe*, we isolated a hypomorphic allele of *spt6*, designated *spt6-K20*, in which a 246-bp region corresponding to the YqgFc RNase H-like domain^14^ of Spt6 has been deleted in-frame (Fig. 1a, Supplementary Figs. 1a–f and 2a–h, see Supplementary Methods for details). We found that *spt6-K20* cells grew slower than wild-type cells (Supplementary Fig. 2a), whereas the complete deletion of *spt6* (*spt6Δ*) caused a much more severe growth defect. Heterochromatin in fission yeast is marked by both Lys-9 methylation of histone H3 and the heterochromatin protein 1 (HP1) homolog Swi6^HP1^ (switch 6), which recognizes Lys-9 methylated histone H3^20^. Insertion of *ade6* in the pericentromere as a marker gene leads to passive construction of heterochromatin at the marker gene by spreading of heterochromatin marks from the native regions^21^. Chromatin immunoprecipitation (ChIP) followed by quantitative polymerase chain reaction (ChIP-qPCR) analysis for dimethylation of Lys-9 in histone H3 (K9me2) and Swi6^HP1^ demonstrated that placement of these heterochromatin-specific marks in the pericentromere (*ade6* and *dg*) and subtelomere (*tlh*) depends solely on the Suv39h family Lys-9-specific methyltransferase Clr4^Suv39h^ (cryptic loci regulator 4)^20^ (Fig. 1b, c).

The importance of fission yeast Spt6 in heterochromatin organization was previously studied with the *spt6-1* mutant allele^17^. Cells carrying the *spt6-1* mutant allele have been shown not to affect K9me2 in the pericentromeric repeats. However, we found that both the *spt6-K20* and *spt6Δ* mutations caused a significant decrease in the levels of K9me2 and Swi6^HP1^ (Fig. 1b, c). In *spt6Δ* cells, some K9me2 remained in the native regions (*dg* and *tlh*), whereas the modification was completely abrogated in the inserted marker gene (*ade6*) (Supplementary Fig. 3a, showing K9me2 level per histone H3 molecule). Therefore, Spt6 is essential for the maintenance of K9me2, especially in the inserted marker gene. Lys-9 methylation of histone H3 is coupled with generation of small interfering RNA (siRNA)^22^,^23^. Consistent with the substantial decrease in the K9me2 level, siRNA corresponding to the pericentromeric repeats was undetectable in *spt6* mutant cells (Supplementary Fig. 4).

To examine the effect of *spt6Δ* on Pol II and histone H3 occupancy across the pericentromeric regions, we performed a ChIP analysis followed by high-throughput sequencing (ChIP-seq) of Pol II and histone H3. The Pol II occupancy in some pericentromeric cryptic regions was dramatically increased in *spt6Δ* cells, as was also shown in *clr4Δ*^Suv39h^ cells^24^ (Fig. 1d). Transcriptional activation in *clr4Δ*^Suv39h^ cells did not lead to an overall decrease in histone H3 occupancy, except in several sensitive regions, as previously reported (Fig. 1d, red asterisks)^25^. It has been shown that the *spt6-1* mutation does not cause a decrease in nucleosome occupancy in the pericentromere^17^. In *spt6Δ* cells, however, a significant decrease in histone H3 occupancy over the cryptic transcribed regions was observed (Fig. 1d and Supplementary Fig. 3b). ChIP-qPCR and micrococcal nuclease treatment followed by qPCR (MNase-qPCR) analyses confirmed that nucleosome loss did occur in the cryptic transcribed regions (Supplementary Fig. 5a–c). Even in a region where Pol II occupancy was very low (Supplementary Fig. 3b, region *dh-L*), a 2-fold increase in Pol II occupancy was associated with a significant decrease in the levels of histone H3 occupancy (Supplementary Fig. 3c) and K9me2 (Supplementary Fig. 3a). Thus, in the absence of Spt6, nucleosome loss and a reduction in the level of K9me2 occur concomitantly in the cryptic transcribed regions in the pericentromere. Note that nucleosome loss did not appear to occur in some subregions within the cryptic transcribed regions in *spt6Δ* cells (Fig. 1d, blue asterisks). These subregions might be resistant to the cotranscriptional nucleosome loss caused by Spt6 inactivation.

### Transcription-coupled nucleosome loss and compensatory deposition of Lys-56 acetylated histone H3 occur in *spt6* mutant cells

**Figure 2:**
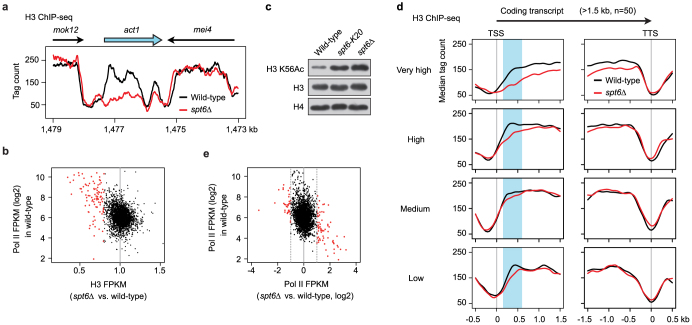
Cotranscriptional nucleosome loss and increased deposition of Lys-56 acetylated histone H3 in *spt6Δ* cells. (a) Occupancy of histone H3 around the *act1* locus. Normalized tag counts at positions from the left end of chromosome II are shown. Arrows indicate the direction and length of transcripts. (b) Comparison of histone H3 occupancy with respect to Pol II occupancy in wild-type cells. Genes with significant loss of histone H3 (<0.8-fold remaining) in *spt6Δ* cells are highlighted in red. FPKM: fragments per kilobase of transcribed region per million mapped reads. (c) Western blotting analysis of histones. Triton X-insoluble fractions extracted from equal numbers of cells were separated by SDS-PAGE and probed with indicated antibodies. The gels have been run under the same experimental conditions. Full-length blots are presented in Supplementary Figure 10. (d) Occupancy of histone H3 around TSS and TTS. Regions corresponding to the first few nucleosomes are highlighted in light blue. (e) Comparison of Pol II occupancy with respect to its level in wild-type cells. Genes with a significant change in Pol II occupancy (>2-fold change) in *spt6Δ* cells are highlighted in red.

Next, we studied the function of Spt6 in euchromatin. Partial inactivation of Spt6 causes nucleosome loss in actively transcribed genes^11^,^17^; therefore, we performed ChIP-seq and MNase-qPCR analyses to examine the effect of complete inactivation of Spt6 on nucleosome occupancy. As expected, significant nucleosome loss over the actively transcribed *act1* gene was observed in *spt6Δ* cells (Fig. 2a and Supplementary Fig. 6). Genome-wide analysis of protein occupancy per gene revealed that the extent of the decrease in histone H3 occupancy in *spt6Δ* cells was greater in genes with higher Pol II occupancy (Fig. 2b), indicating that transcription induces nucleosome loss. In the naked regions from which pre-existing histone molecules have been dissociated, it is possible that nucleosomes could be reconstructed with other histone molecules. Newly synthesized histone H3, which does not carry “locus-specific” posttranslational modifications, is acetylated at Lys-56 (K56Ac) before deposition^9^,^26^,^27^. Therefore, this modification serves as a marker for newly deposited histone H3^28^,^29^. As shown in Figure 2c, Western blotting analysis of bulk histones in the chromatin fraction revealed that the amounts of histones H3 and H4 do not change dramatically, and that the level of Lys-56 acetylated histone H3 (K56Ac) was apparently increased in *spt6* mutant cells (Fig. 2c). These results strongly suggest that the cotranscriptional decrease in histone H3 occupancy is partially compensated for by Spt6-independent deposition of Lys-56 acetylated histone H3.

To examine histone H3 occupancy with respect to transcription start sites (TSS) and termination sites (TTS), we analyzed four sets of genes transcribed at different levels (“Very high”, “High”, “Medium”, and “Low”) (Supplementary Fig. 7a, b, see Supplementary Methods for details). Median histone occupancy was slightly lower in the “Very high” and “Low” genes than in the “High” and “Medium” genes in wild-type cells (Fig. 2d and Supplementary Fig. 7c). In *spt6Δ* cells, the decrease in histone H3 occupancy was indeed greater in genes with higher transcription levels (Fig. 2d and Supplementary Fig. 8). In contrast to previous studies in which Spt6 was not completely depleted^11^, in our study a significant decrease in histone H3 occupancy was observed even in the “Low” genes (Fig. 2d and Supplementary Fig. 8). The weaker effect in the “Low” genes may reflect Spt6-independent deposition of K56Ac-marked histones that acts antagonistically to the cotranscriptional nucleosome loss. We noted that Pol II occupancy in genes with low expression levels was increased in *spt6Δ* cells (Fig. 2e). Consistently, the levels of both sense and antisense transcripts with low expression levels in wild-type cells were increased in *spt6Δ* cells (Supplementary Fig. 9). Therefore, despite the observation that Spt6-independent deposition can partially compensate for the decrease in nucleosome occupancy, such quantitative compensation may be insufficient for the maintenance of the epigenetically repressed state. Histone H3 occupancy was decreased predominantly in the region corresponding to the first few nucleosomes (Fig. 2d and Supplementary Fig. 8, highlighted in light blue), suggesting that either nucleosome loss predominantly occurs in this region or that compensatory deposition rarely occurs here.

### Spt6 prevents transcription-coupled loss of Lys-4 methylated histone H3

**Figure 3:**
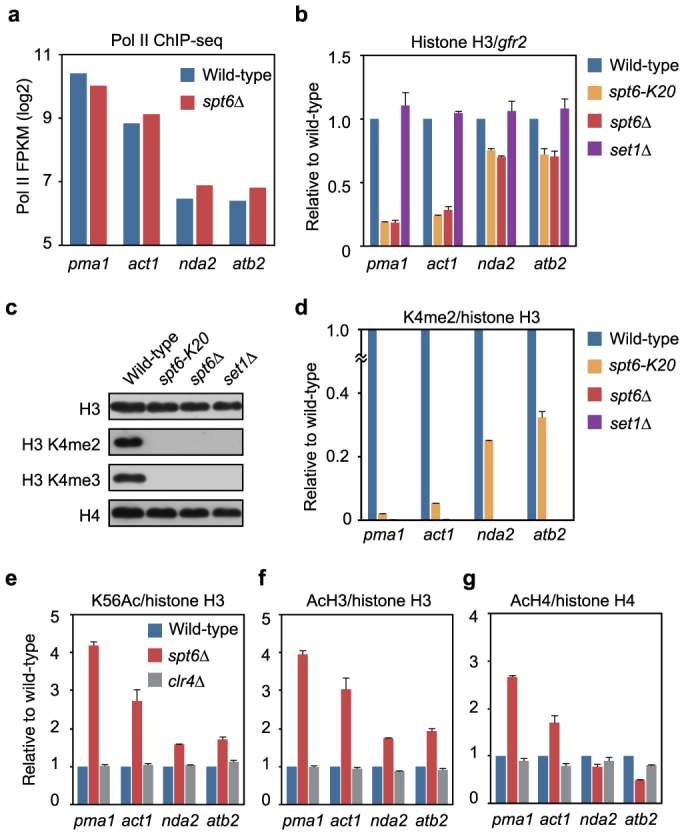
Spt6 is essential for the maintenance of Lys-4 methylated histone H3. (a) Pol II occupancy of indicated genes is shown as FPKM. (b) ChIP-qPCR analysis of histone H3. Histone H3 levels in the indicated genes are normalized to the gene-free region *gfr2*. Fold-enrichment relative to wild-type cells is shown. (c) Western blotting analysis of histones and histone H3 modifications. Samples were prepared as described in Fig. 2c and probed with the antibodies indicated. The gels have been run under the same experimental conditions. Full-length blots are presented in Supplementary Figure 10. (d–g) ChIP-qPCR analysis of the level of K4me2 (d), K56Ac (e), AcH3 (f), and AcH4 (g) in the indicated genes. The y-axis in (d) is magnified for comparison. Error bars, s.d.

Newly deposited histone H3 molecules may not carry locus-specific posttranslational modifications. Therefore, the next question we sought to answer was whether or not inactivation of Spt6 facilitates cotranscriptional elimination of histone methylation in coding genes. We focused on four housekeeping genes in which Pol II occupancy was not dramatically altered by Spt6 inactivation (Fig. 3a). ChIP-qPCR analysis demonstrated that the extent of the decrease in histone H3 occupancy in *spt6* mutant cells correlated with Pol II occupancy (Fig. 3a, b). In fission yeast, the Lys-4 residue of histone H3 is methylated solely by the Set1-family methyltransferase Set1^30^,^31^. Western blotting analysis of bulk histones demonstrated the necessity of Set1 for di- and trimethylation at Lys-4 of histone H3 (K4me2 and K4me3, respectively) (Fig. 3c). In *spt6* mutant cells, K4me2 and K4me3 could not be detected (Fig. 3c). ChIP-qPCR analyses consistently revealed that the level of K4me2 decreased significantly in *spt6-K20* cells and was undetectable in *spt6Δ* cells (Fig. 3d; Note: the y-axis is magnified). The significant decrease in the level of K4me2 suggests that either Spt6 mediates Set1-dependent placement of Lys-4 methylation in histone H3, or that Spt6 retains Lys-4 methylated histone H3 during transcription. If the former is true, partial inactivation of Spt6 should attenuate Set1 activity, leading to a uniform reduction in the level of K4me2 in each gene. Importantly, K4me2 levels in *spt6-K20* cells were lower in genes with higher Pol II occupancy (Fig. 3a, d). In *spt6Δ* cells, the levels of K56Ac and acetylation of histone H3 at Lys-9 and Lys-14 (AcH3) were higher in genes with higher Pol II occupancy (Fig. 3a, e, f). The level of histone H4 acetylation (AcH4) was also altered in *spt6Δ* cells (Fig. 3g). In contrast, these histone acetylation marks were not altered in the heterochromatin-defective *clr4Δ*^Suv39h^ cells (Fig. 3e–g). These data suggest that inactivation of Spt6 causes cotranscriptional histone turnover, which in turn leads to impaired histone acetylation. Both the negative correlation between the levels of K4me2 and Pol II occupancy and the cotranscriptional histone turnover in *spt6* mutant cells strongly suggest that transcription *per se* induces the loss of histones with locus-specific modifications when Spt6 does not function appropriately.

### Spt6 acts in parallel with Clr3^HDAC^ to maintain heterochromatin in the pericentromere

**Figure 4:**
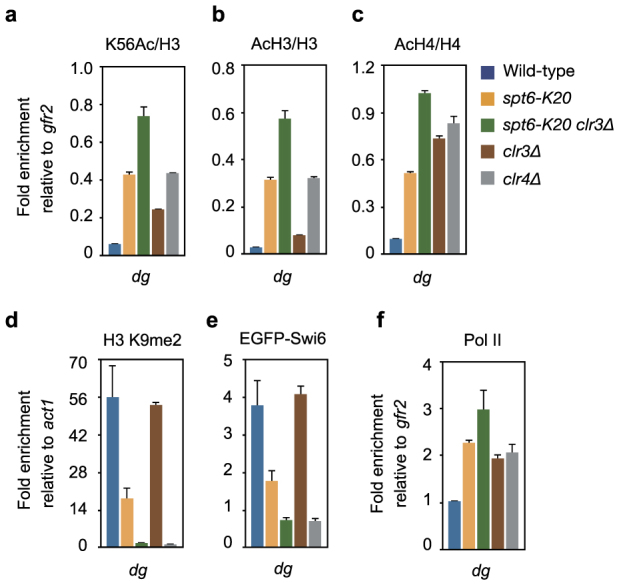
Spt6 acts in parallel with Clr3 histone deacetylase to maintain heterochromatin in the pericentromere. (a–f) ChIP-qPCR analysis of K56Ac (a), AcH3 (b), AcH4 (c), K9me2 (d), EGFP-Swi6^HP1^ (e), and Pol II (f) in the pericentromeric *dg* repeat. Error bars, s.d.

Recent studies have shown that heterochromatic silencing in fission yeast is maintained in part by a Clr3^HDAC^-mediated prevention of histone turnover^18^,^25^,^32^,^33^. In *spt6* mutant cells, the majority of residual histone H3 in the pericentromere was not methylated at Lys-9 (Supplementary Fig. 3a), suggesting that the loss of Lys-9 methylation occurs as a result of cotranscriptional histone turnover. Therefore, we examined the relationship between Spt6 and Clr3^HDAC^ genetically. ChIP-qPCR analysis showed that the levels of K56Ac in the pericentromere in both *clr3Δ*^HDAC^ and *clr4Δ*^Suv39h^ single mutant cells were higher than in wild-type cells (Fig. 4a), demonstrating that histone turnover is prevented by these factors^18^. The K56Ac level in the pericentromere was also increased in *spt6-K20* single mutant cells (Fig. 4a).

In order to determine whether Spt6 acts in the Clr3^HDAC^-dependent prevention of histone turnover, we combined the *spt6-K20* mutation with *clr3Δ*^HDAC^. In the *spt6-K20* *clr3Δ*^HDAC^ double mutant cells, the K56Ac level in the pericentromere was higher than that in each of the single mutant cells (Fig. 4a), suggesting that Spt6 and Clr3^HDAC^ have redundant mechanisms to prevent histone turnover. The increase in the K56Ac level was associated with an increase in the acetylation level of the N-terminal tail of both histone H3 (AcH3) and histone H4 (AcH4) (Fig. 4b, c). In addition, the levels of K9me2 and Swi6^HP1^ were further decreased in the double mutant cells to levels comparable to those in *clr4Δ*^Suv39h^ cells (Fig. 4d, e). Consistently, Pol II occupancy, which was higher in each of the single mutant cells, was increased further in the double mutant cells (Fig. 4f). The additive effect observed here suggests that Spt6 acts in parallel with Clr3^HDAC^ to prevent histone turnover and the associated subsequent loss of histone H3 Lys-9 methylation and derepression of cryptic transcription.

## Discussion

**Figure 5:**
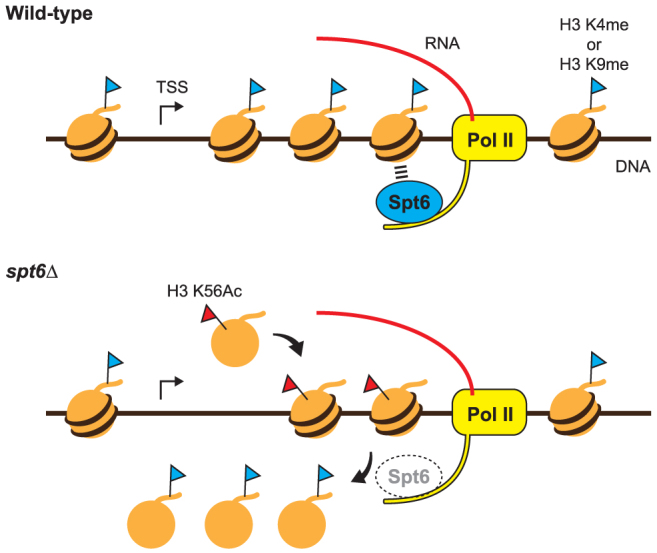
Model describing Spt6-dependent retention of epigenetically modified histones during transcription. In wild-type cells, Pol II transcribes the nucleosome templates, keeping histones H3 and H4 in its wake. The histone H3–H4 chaperone Spt6 interacts directly with the phosphorylated C-terminal domain of elongating Pol II. Spt6 retains posttranslationally modified histone H3 during transcription so that the state of the chromatin is maintained. In the absence of Spt6 activity (*spt6Δ*), modified histones are displaced during transcription. Spt6-independent deposition of K56Ac-marked histones partially compensates for the loss of nucleosome occupancy. However, as the newly deposited histones do not carry appropriate locus-specific modifications, epigenetic control of the transcribed region is impaired.

Our data indicate that when Pol II lacking a functional Spt6 transcribes through the nucleosome template, nucleosome loss occurs in the wake of Pol II and eventually leads to Spt6-independent deposition of histones without locus-specific modifications. Therefore, we propose that during transcription, the histone H3–H4 chaperone Spt6 retains histone H3 to keep the rate of transcription-coupled histone turnover to a minimum in order to maintain locus-specific posttranslational modifications (Fig. 5). Alternatively, the transcription-coupled loss of histone modifications could result from an error in the recruitment of histone modification enzymes to the transcribed regions that is potentially mediated by Spt6. However, transcription-coupled nucleosome loss and increased acetylation of histone H3 at Lys-56 in the *spt6* mutant cells support the idea that elimination of locus-specific histone modifications occurs as a result of histone turnover. The precise mechanism of Spt6-dependent maintenance of histone modifications should be studied at the molecular level in the future.

The essentiality of Spt6 for the maintenance of histone positioning and associated modifications suggests that intracellular mechanisms exist that inactivate Spt6 in *cis* to reset the chromatin state or create accessible chromatin^34^. As almost all of the genome is transcribed to some extent^35^, inactivation of Spt6 could cause a catastrophic breakdown in epigenomic integrity. Furthermore, Spt6 breakdown in human cells could be involved in the epigenetic alterations frequently observed in cancer cells^36^.

## Methods

### Genetic manipulations

A fission yeast wild-type strain was mutagenized with 254-nm ultraviolet light to obtain mutants defective in pericentromeric silencing. The corresponding mutation was identified by whole-genome sequencing.

### Chromatin immunoprecipitation

Exponentially growing cells were subjected to chromatin immunoprecipitation (ChIP). For ChIP-qPCR analysis of histone modifications, the immunoprecipitation efficiency of K4me2, K9me2, K56Ac, AcH3 and AcH4 at each locus was first normalized to that of the C-terminal region of histone H3 or histone H4 (for AcH4) in order to evaluate the level of modification per histone molecule. The modification levels were then compared to those in the wild-type control (y-axis: relative to wild-type) or the internal control regions indicated. For ChIP-qPCR analysis of K9me2 and Swi6 in Figures 1 and 4, the immunopreciptation efficiency at each locus was compared to those in the *act1* region. For ChIP-seq analysis, approximately 10 million single-end 49-base reads were mapped onto the 972 reference genome. Pileup data generated by MACS^37^ were analyzed using the R-statistical environment (http://www.R-project.org). The R-scripts used in this study are available upon request.

Full Methods and any associated references are available as Supplementary Information.

### Accession codes

High-throughput sequencing data in this publication have been deposited in DRA (http://trace.ddbj.nig.ac.jp/dra) under accession numbers DRA000544, DRA000855, DRA000856, and DRA000857. Microarray data are available in the ArrayExpress database (www.ebi.ac.uk/arrayexpress) under accession number E-MTAB-1373. Contig sequences have been deposited in the DDBJ (http://www.ddbj.nig.ac.jp/) under accession numbers AB762285 and AB762286.

## Author Contributions

T.I. performed the northern blotting analysis; J.N. generated the *egfp-swi6*^+^ strain; K.O. and H.K. performed experiments and analyzed the data; Y.M. and T.U. were involved in study design; and H.K. designed the study, wrote the R-scripts used to analyze the data, and wrote the manuscript. All authors discussed the results and commented on the manuscript.

## Supplementary Material

Supplementary Information Supplementary Information
