# Supplementary material for: Spt6 prevents transcription-coupled loss of posttranslationally modified histone H3
Source: Sci Rep. 2013 Jul 15;3:2186. doi: 10.1038/srep02186 (PMC3711048; doi:10.1038/srep02186)
Supplement: Supplementary Information [file srep02186-s1.pdf]

Supplementary Information

Spt6 prevents transcription-coupled loss of posttranslationally modified histone H3

Hiroaki Kato[1,2]*, Kosuke Okazaki[1], Tetsushi Iida[2,3], Jun-ichi Nakayama[4,5], Yota Murakami[6] and Takeshi Urano[1]

[1]Department of Biochemistry, Shimane University School of Medicine, Izumo, Japan
[2]PRESTO, Japan Science and Technology Agency (JST), Saitama, Japan
[3]Division of Cytogenetics, National Institute of Genetics, Mishima, Japan
[4]Graduate School of Natural Sciences, Nagoya City University, Nagoya, Japan
[5]Laboratory for Chromatin Dynamics, RIKEN Center for Developmental Biology, Kobe, Japan
[6]Laboratory of Bioorganic Chemistry, Department of Chemistry, Faculty of Science, Hokkaido University, Sapporo, Japan

*Correspondence to: Hiroaki Kato
Department of Biochemistry, Shimane University School of Medicine
89-1 Enya-cho, Izumo 693-8501, Japan
Tel: +81-853-20-2126; Fax: +81-853-20-2125
E-mail: hkato@med.shimane-u.ac.jp

1. Supplementary Figures and Legends:

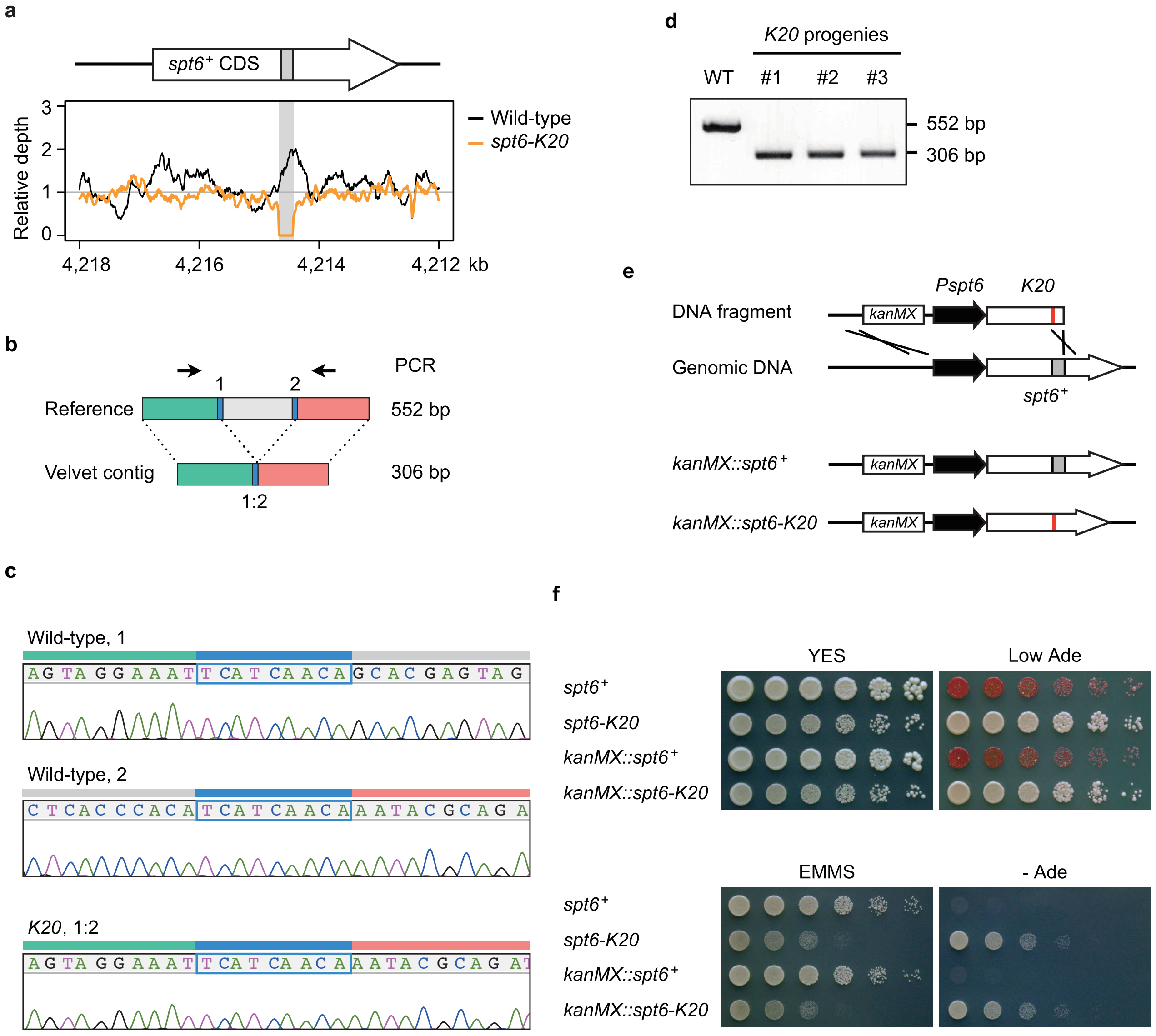

Supplementary Figure 1. Isolation of *spt6-K20* as a silencing mutant

(a) Sequence reads that were prepared from the original *K20* strain were not mapped to a region in the *spt6*$^{+}$ gene. Positions from the left end of chromosome II are shown on the x-axis. Mapping depth at each position was divided by the mean depth and is shown on the y-axis as relative depth. Arrow indicates the coding sequence of *spt6*$^{+}$. The unmapped (deleted) region is shaded in gray. (b) A contig produced by Velvet had a 246-bp deletion in the *spt6* gene. Arrows indicate positions of primers for PCR. The size of each PCR product is shown. The left (green) and right (pink) regions flanking the deleted region (gray) are shown. Blue boxes indicate the 9-bp elements (TCATCAACA) flanking the inner 237-bp

sequence. (c) Results of Sanger sequencing for the left (wild-type, 1) and right (wild-type, 2) regions flanking the deleted region. The junction in *spt6-K20* is shown (*K20*, 1:2). Horizontal bars (green, blue, gray, and pink) indicate the like-colored regions in (b). (d) PCR analysis of the deleted region. Genomic DNAs purified from wild-type (WT) and *K20* progenies were used as templates. The PCR products were separated on a 2% agarose gel, which was then stained with ethidium bromide. (e) Schematic representation of confirmation of the *K20* phenotype by gene recombination. A DNA fragment containing a *kanMX* marker gene and the *K20* deletion was replaced with the genomic *spt6*$^{+}$ gene. Black arrows indicate the *spt6*$^{+}$ promoter region (*Pspt6*). White arrows indicate the *spt6*$^{+}$ coding region. The region corresponding to the deleted region is shown in gray. The *K20* deletion is indicated as a vertical red line. In addition to the strain that possessed the *K20* deletion (*kanMX::spt6-K20*), a G418-resistant strain with the wild-type *spt6*$^{+}$ gene (*kanMX::spt6*$^{+}$) was generated as the wild-type control. (f) Serial dilution plating assays used to examine gene silencing of *otr1R::ade6*$^{+}$. Cells were spotted onto indicated plates and incubated at 30˚C for 4 days.

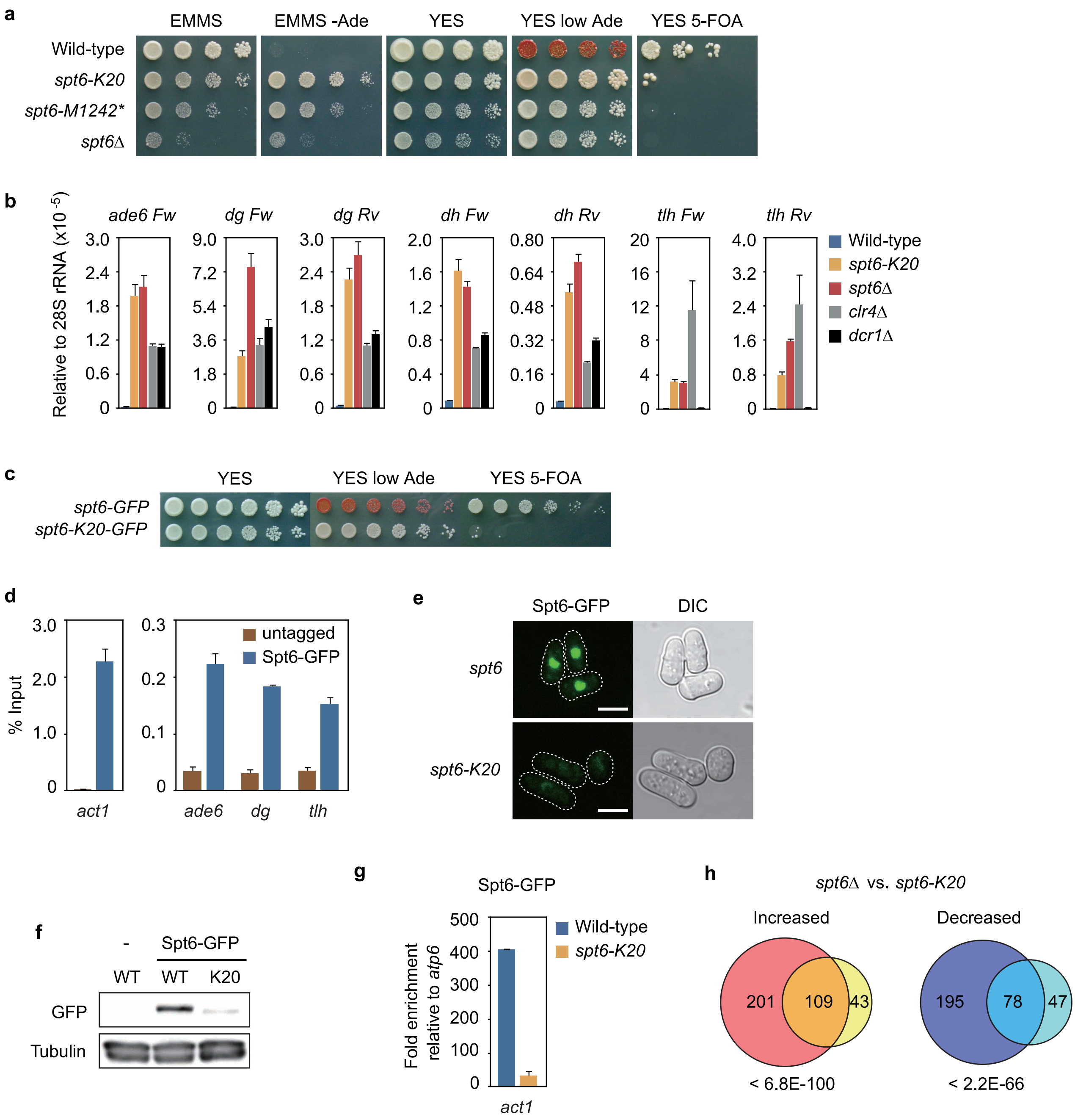

Supplementary Figure 2. *spt6-K20* is a hypomorphic mutant

(a) Serial dilution plating assays used to examine silencing of the *otr1R::ade6*$^{+}$ and *imr1L::ura4*$^{+}$ genes. Cells were spotted onto indicated plates and incubated at 30˚C for 4 days. (b) Strand-specific RT-qPCR analysis of heterochromatic transcripts. The concentration of each transcript relative to the forward strand of 28S ribosomal RNA is shown on the y-axis. (c) Serial dilution plating assays as in (a). (d) ChIP-qPCR analysis of

Spt6-green fluorescent protein (GFP). The efficiency of immunoprecipitation is shown as the percentage of input DNA on the y-axis. (e) Fluorescent microscopic observation of Spt6-GFP (bar = 5 μm). DIC; differential interference contrast microscopy. (f) Western blotting analysis of Spt6-GFP. Protein extracts were separated by SDS-PAGE and detected with antibodies against GFP and tubulin. (g) ChIP-qPCR analysis of Spt6-GFP. Fold enrichment relative to the mitochondrial *atp6* gene is shown on the y-axis. (h) Significant overlap of dysregulated genes in *spt6*Δ and *spt6-K20* cells. Genes showing a significant increase or decrease (>1.5-fold, $P<0.05$, Student's t-test) in expression in each strain were compared. *P*-values (Bonferroni corrected Fisher's exact test) are shown under the Venn diagrams. Error bars, s.d.

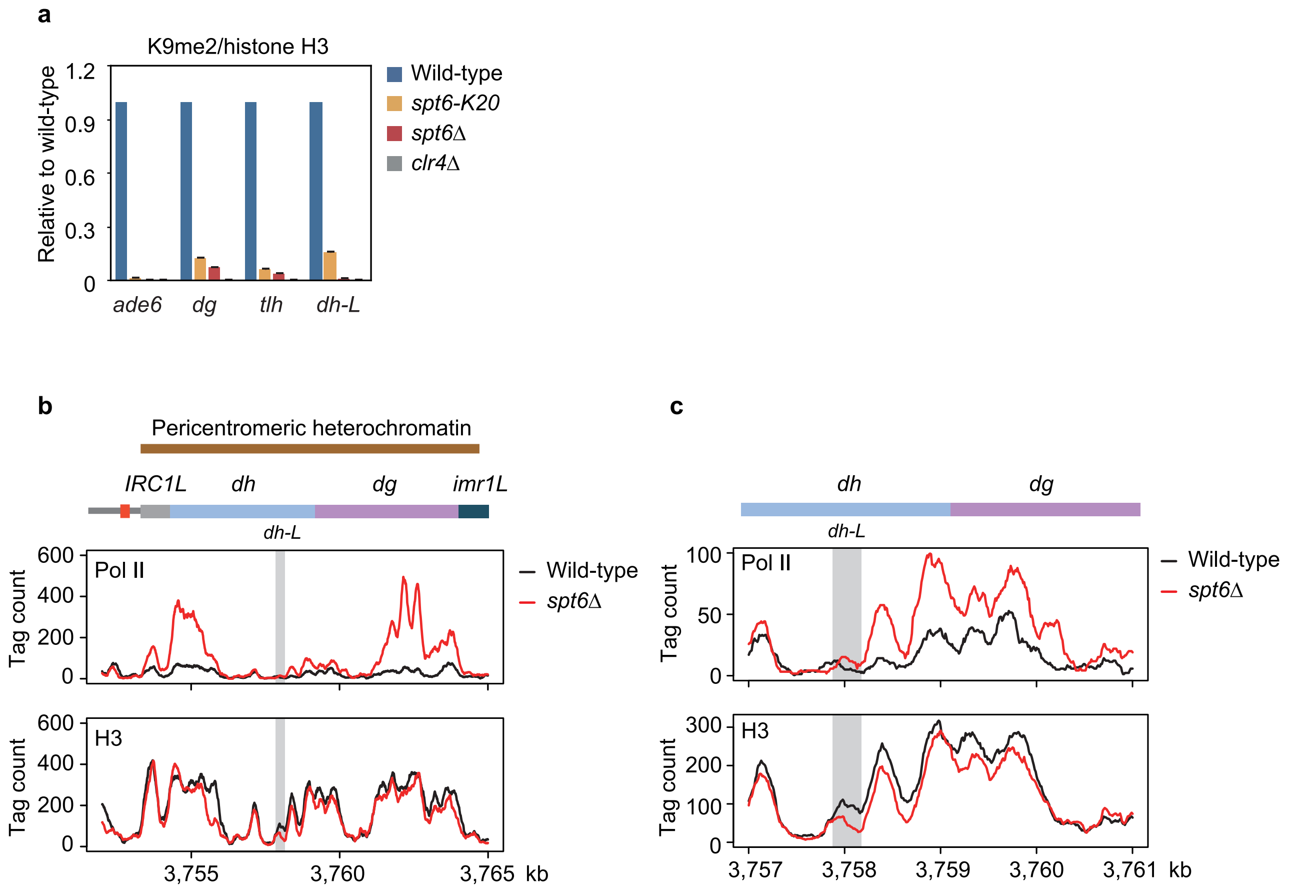

Supplementary Figure 3. Cotranscriptional loss of K9me2 in *spt6* mutants

(a) ChIP-qPCR analysis of the K9me2 level per histone H3 molecule. Error bars, s.d. (b) ChIP-seq analysis of Pol II and histone H3 around the left side of centromere 1 (see Fig. 1d). Tag count of Pol II and histone H3 for each strain is shown. The *dh-L* subregion is highlighted in gray. (c) Magnified view of (b) showing detail of Pol II and histone H3 occupancy in the *dh-L* subregion.

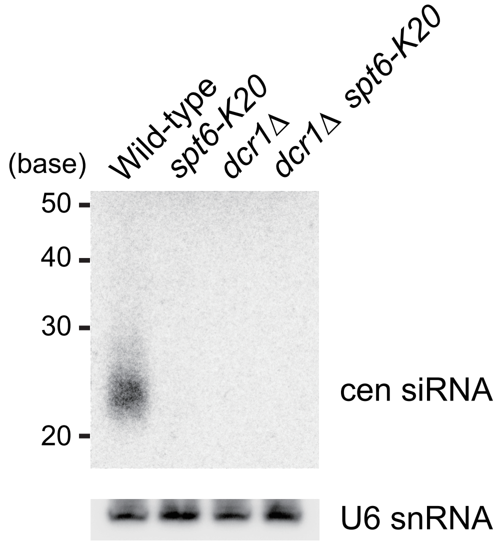

Supplementary Figure 4. Northern blotting of pericentromeric siRNA

U6 RNA was used as a loading control for RNA quantity. *dcr1*Δ^{Dicer} cells, which are defective in siRNA generation, were used as a negative control.

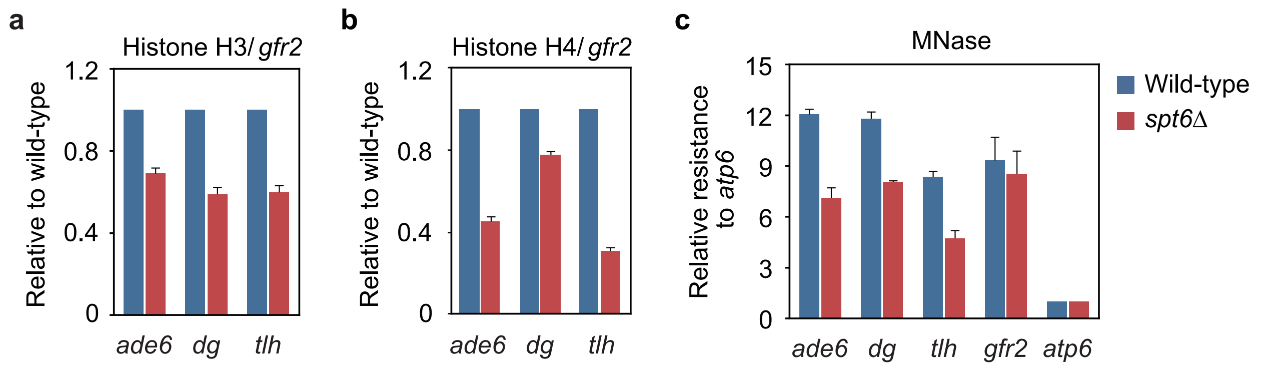

Supplementary Figure 5. Spt6 prevents nucleosome loss in heterochromatin

(a, b) ChIP-qPCR analysis of histones H3 (a) and H4 (b). Histone levels in indicated genes are normalized to the gene-free region *gfr2*. Fold enrichment relative to wild-type cells is shown on the y-axis. (c) MNase-qPCR analysis. MNase-treated DNA was purified and quantified by qPCR. The ratio of MNase-treated DNA to mock-treated DNA, normalized to the mitochondrial non-nucleosomal *atp6* gene, is shown as relative resistance to *atp6*. Note that the MNase resistance in the *grf2* region is not changed in *spt6*Δ cells. Error bars, s.d.

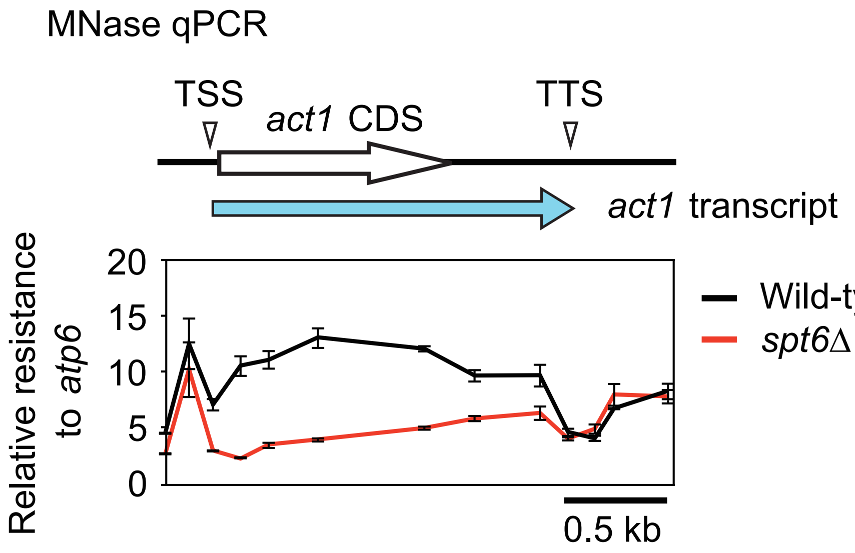

Supplementary Figure 6. MNase-qPCR analysis of the *act1* gene

MNase-treated DNA was purified and quantified by qPCR. The ratio of MNase-treated DNA to mock-treated DNA, normalized to the mitochondrial non-nucleosomal *atp6* gene, is shown as relative resistance to *atp6*. Error bars, s.d.

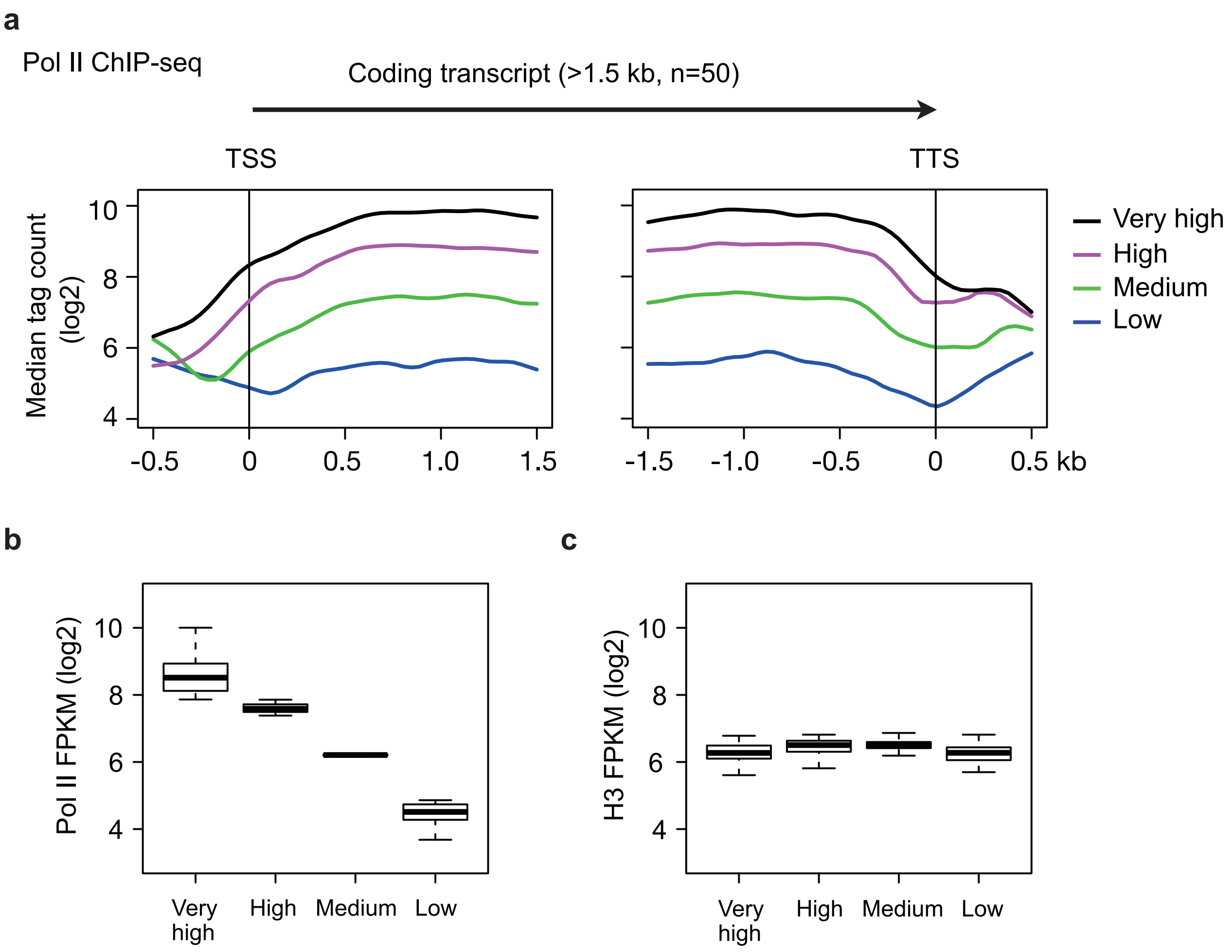

Supplementary Figure 7. Four gene sets with different expression levels

(a) ChIP-seq analysis of Pol II. Position (kb) relative to the transcription start site (TSS) and termination site (TTS) is shown on the x-axis. Median tag counts of indicated gene sets, which are transcribed at different levels, are shown on the y-axis. (b, c) Distributions of the FPKM for Pol II (b) and histone H3 (c) in indicated gene sets. Boxplots show the median, quartiles, maximum, and minimum FPKM in each gene set.

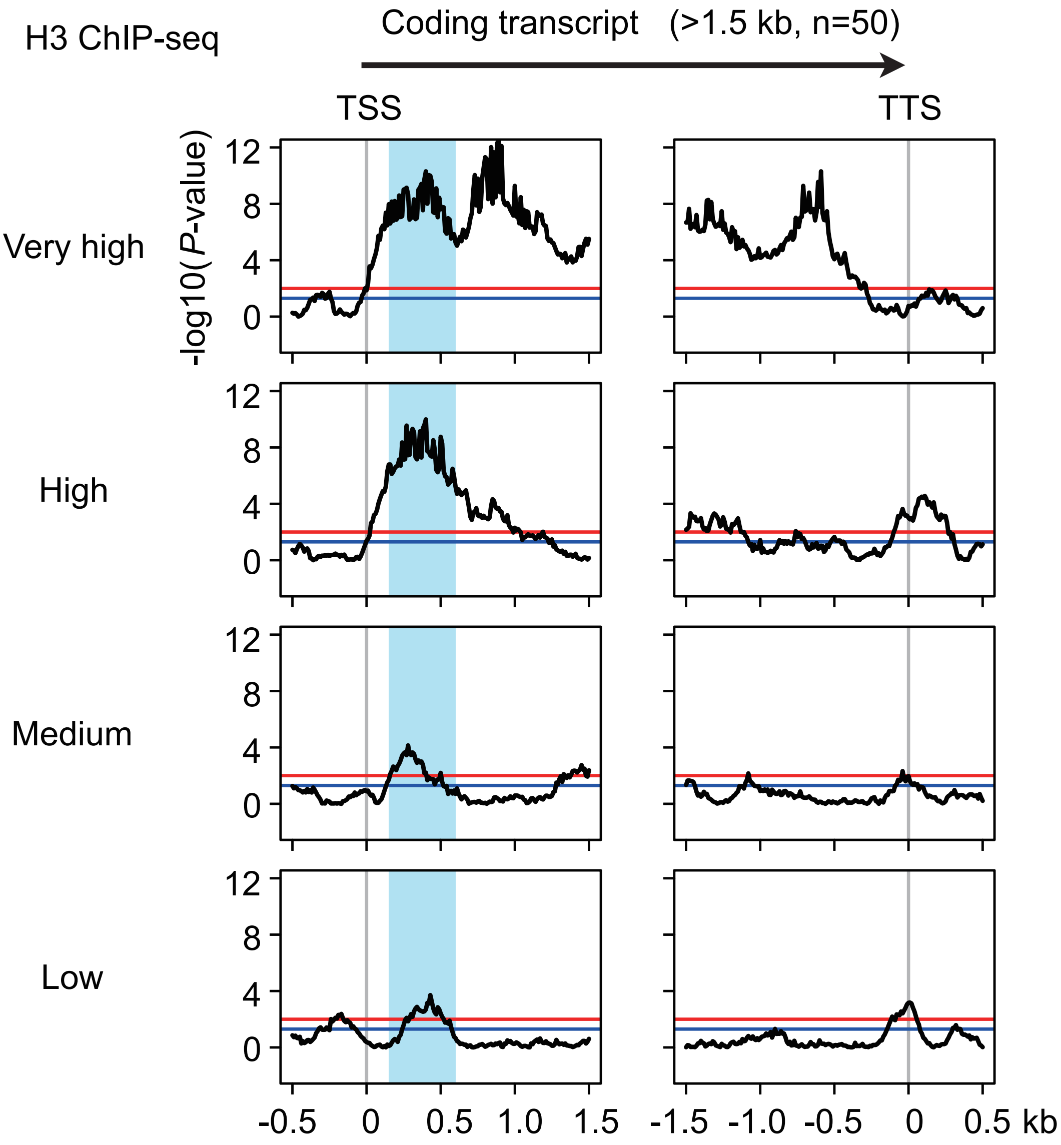

Supplementary Figure 8. Statistical analysis of histone H3 ChIP-seq data

Position (bp) relative to TSS and TTS is shown on the x-axis. *P*-values (Wilcoxon's signed rank test, n = 50) for each interval in Fig. 2d are shown on the y-axis. Regions corresponding to the first few nucleosomes are highlighted in light blue. Horizontal bars in red (*P*-value=0.01) and blue (0.05) indicate the threshold.

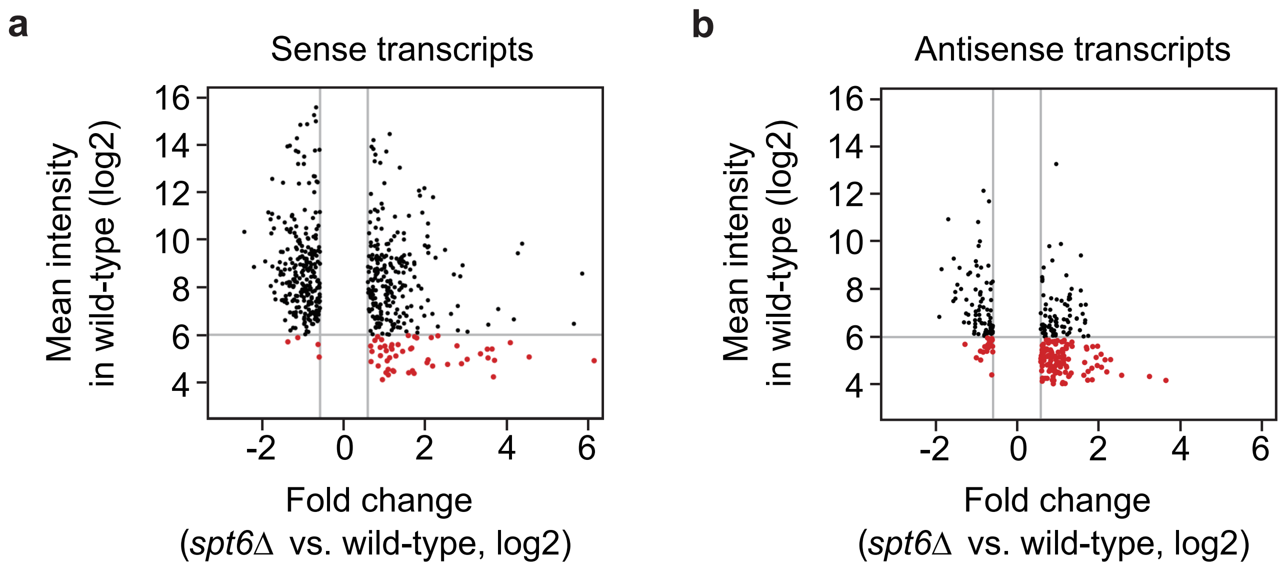

Supplementary Figure 9. Transcripts of low-expression-level genes in wild-type cells were increased in *spt6*Δ cells

Microarray-based transcriptome analysis of sense (a) and antisense (b) transcripts. Transcripts that showed significant change (>1.5-fold, $P<0.05$, Student's t-test) in *spt6*Δ cells are plotted. The ratio of mean transcript intensity in mutant cells to that in wild-type cells is shown as fold change on the x-axis. Mean transcript intensity in wild-type cells is shown on the y-axis. Genes with relatively lower intensity in wild-type cells are highlighted in red.

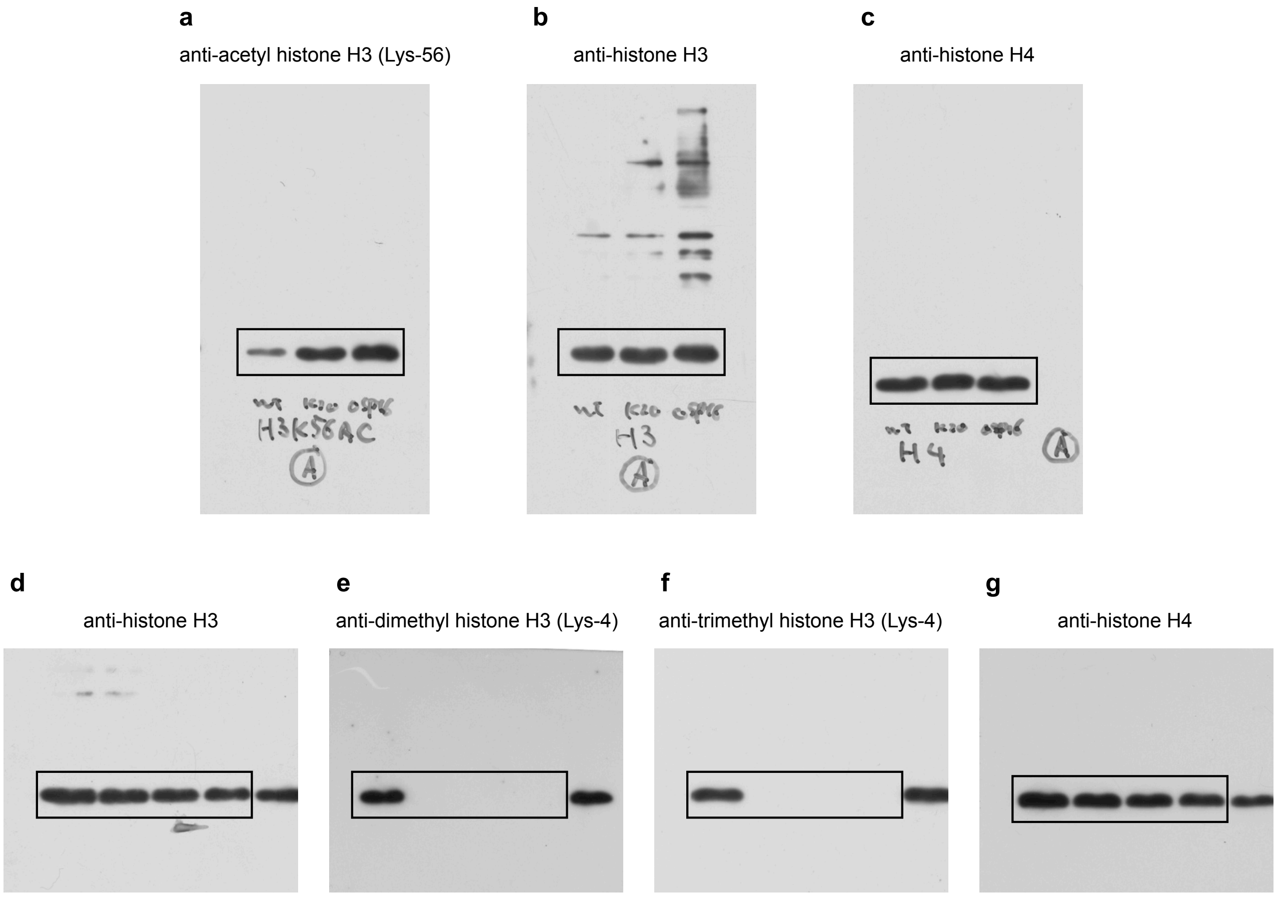

Supplementary Figure 10. Full-length blot images for Figures 2c and 3c

(a-c) Western blot images for K56Ac (a), histone H3 (b) and histone H4 (c) in Figure 2c. (d-g) Western blot images for histone H3 (d), K4me2 (e), K4me3 (f) and histone H4 (g) in Figure 3c.

2. Supplementary Tables:

Supplementary Table 1. Fission yeast strains used in this study

Strain	Genotype	Reference
HKM-35	*h^-, ade6Δ::kanMX, ura4-DS/E, imr1L::$ura4^+$, otr1R::$ade6^+$*	[19]
HKM-1269	*h^-, ade6Δ::kanMX, ura4-DS/E, imr1L::$ura4^+$, otr1R::$ade6^+$, spt6-K20*	This study
HKM-1100	*h^-, ade6-DN/N, ura4-DS/E, imr1L::$ura4^+$, otr1R::$ade6^+$*	This study
HKM-1376	*h^-, ade6-DN/N, ura4-DS/E, imr1L::$ura4^+$, otr1R::$ade6^+$, spt6-K20*	This study
HKM-1715	*h^-, ade6-DN/N, ura4-DS/E, imr1L::$ura4^+$, otr1R::$ade6^+$, spt6-M1242*-kanMX*	This study
HKM-1220	*h^-, ade6-DN/N, ura4-DS/E, imr1L::$ura4^+$, otr1R::$ade6^+$, clr4Δ::hphMX*	This study
HKM-1567	*h^-, ade6-DN/N, ura4-DS/E, imr1L::$ura4^+$, otr1R::$ade6^+$, kanMX::$spt6^+$*	This study
HKM-1617	*h^-, ade6-DN/N, ura4-DS/E, imr1L::$ura4^+$, otr1R::$ade6^+$, kanMX::spt6-K20*	This study
HKM-1556	*h^-, ade6-DN/N, ura4-DS/E, imr1L::$ura4^+$, otr1R::$ade6^+$, egfp-$swi6^+$*	This study
HKM-1779	*h^-, ade6-DN/N, ura4-DS/E, imr1L::$ura4^+$, otr1R::$ade6^+$, egfp-$swi6^+$, clr3Δ::hphMX*	This study
HKM-1681	*h^-, ade6-DN/N, ura4-DS/E, imr1L::$ura4^+$, otr1R::$ade6^+$, egfp-$swi6^+$, clr4Δ::hphMX*	This study
HKM-1642	*h^-, ade6-DN/N, ura4-DS/E, imr1L::$ura4^+$, otr1R::$ade6^+$, egfp-$swi6^+$, spt6-K20*	This study
HKM-1809	*h^-, ade6-DN/N, ura4-DS/E, imr1L::$ura4^+$, otr1R::$ade6^+$, egfp-$swi6^+$, clr3Δ::hphMX, spt6-K20*	This study
HKM-1957	*h^-, ade6-DN/N, ura4-DS/E, imr1L::$ura4^+$, otr1R::$ade6^+$, egfp-$swi6^+$, spt6Δ::kanMX*	This study
HKM-1850	*h^-, ade6-DN/N, ura4-DS/E, imr1L::$ura4^+$, otr1R::$ade6^+$, egfp-$swi6^+$, dcr1Δ::hphMX*	This study
HKM-1592	*h^-, ade6-DN/N, ura4-DS/E, imr1L::$ura4^+$, otr1R::$ade6^+$, $spt6^+$-gfp::kanMX*	This study
HKM-1716	*h^-, ade6-DN/N, ura4-DS/E, imr1L::$ura4^+$, otr1R::$ade6^+$, spt6-K20-gfp::kanMX*	This study
HKM-157	*h^-,ade6-m210, leu1-32, ura4-D18, set1Δ::kanMX*	Lab stock
HKM-1658	*h^-, ura4-DS/E*	This study
HKM-1630	*h^-, ura4-DS/E, dcr1Δ::hphMX*	This study
HKM-1622	*h^-, ura4-DS/E, spt6-K20*	This study
HKM-1665	*h^-, ura4-DS/E, spt6-K20, dcr1Δ::hphMX*	This study

Supplementary Table 2. Primers used for genetic manipulations

Deletion and C-terminal tagging of *spt6*.

spt6-d1	AAGACAAAGAGCGTGGATGG
spt6-d2	TCGACCTGCAGCGTACGACTATAGTGGACGAAGGAACG
spt6-t1	TCCTACCAACATATCGATGTG
spt6-t2	TCGACCTGCAGCGTACGACCCGCCTCCACCCATCCGTCTAAAATTTTTAGTTCG
spt6-t3	TAAACGAGCTCGAATTCATCGATGAGCATTGATGGTTTAAACGC
spt6-t4	GATTTAAGCACCACAAAGCTC

Introduction of the *K20* deletion into the genome.

spt6-i1	GATACCGTTCCTACTTGCAG
spt6-i2	TCGACCTGCAGCGTACGAAACACGTAAACCTTTTGAGCG
spt6-i3	TAAACGAGCTCGAATTCATCGATCCGTGTAAGTTATTCAAAGGG
spt6-i4	TCAGCTGGATAACCATCCTC
spt6-K20-Fw	CGACTAACGCAGTTTCCAAG
spt6-K20-Rv	CAAGACTTGATCAAGCTCCC

Amplification of the *pFA6a* cassettes.

pFA6a-com5	TCGTACGCTGCAGGTCGA
pFA6a-com6	ATCGATGAATTCGAGCTCGTTTA

Supplementary Table 3. Primers used for RT-, MNase- and ChIP-qPCR

Target	Name	Sequence
28S rRNA	28S-rDNA-Fw	GGGAAGGAATTTCCTCAACC
	28S-rDNA-Rv*	AACCTCACGTTCGGTTCATC
act1	act1-1-Fw	TGGCTCTGGTATGTGCAAAG
	act1-1-Rv	AGCTTCATCACCAACGTAGG
	act1-1.2-Fw	GCAAGCGTGGTATTTTGACC
	act1-1.2-Rv	TCAGTCAACAAGCAAGGGTG
	act1-2-Fw	TACCACTGGTATCGTCTTGG
	act1-2-Rv	TAGTCAGTCAAGTCACGACC
	act1-3-Fw	GGTCAAGATTGTTGCTCCTC
	act1-3-Rv	ACGGTAAACGATACCAGGTC
	act1-4-Fw	TCATGAGGAACTTTGGGTAT
	act1-4-Rv	AAAGGCTAGCTCTGCATTCG
	act1-5-Fw	TACAAGGTGGTAACTGCGAG
	act1-5-Rv	AAGTTATACAAGTTCAGGTACC
	act1-5.2-Fw	GAAGCCAAATGGTTGAATGTG
	act1-5.2-Rv	GAACACTAATCATACCAATGTC
	act1-6-Fw	GACATTGGTATGATTAGTGTTC
	act1-6-Rv	TGGATTTGTTTATACAGAGAGG
	act1-6.2-Fw	CGACGCCTCTCTGTATAAAC
	act1-6.2-Rv	CTCTTGTAATAATATATGTTGACG
	act1-7-Fw	CTCGTGACAGAGTATAGACG
	act1-7-Rv	AGCTGATAACATCCTTTATTCG
	act1-NFR-Fw	ACACAGCGTTAGCACTTCAC
	act1-NFR-Rv	AAGGATTTGCTATATCGCTTAC
	act1-Nuc1-Fw	ATAAGATACACAGTTATACAGTG
	act1-Nuc1-Rv	GTGGCTCAAATATAGCAATTTC
	act1-TSS-Fw	ATCAACGGCTTCATACCACC
	act1-TSS-Rv	ACCAACGCTGCGATTTCTTC
ade6	ade6-wt-Fw	TTGCAGTGATGGTAGTACGC
	ade6-wt-Rv	GAGCACGCTGTTGAATTGAG
atb2	atb2-Fw	CCAAGCAGGAACTCAAATCG
	atb2-Rv	TTTCCTTGACCAGTTTCTGAG
atp6	atp6-Fw	ACACCATATGGATCAGGAGC
	atp6-Rv	ATGGGAAATAGCTTTGTCCTG
dg	dg-Fw	TTATGGCGATGCCAAACAAC
	dg-Rv	AGGATCATCGAGAAGAGTAG
dh	dh-Fw	CACCAGACCATTACAAGCAC
	dh-Rv	CTTCTCTTGAATAAAACCGCC
dh-L	dh-L-Fw	GAGTTGTTATACCTTTCGAGG
	dh-L-Rv	AATCACTTCTGCGACTCATAC
gfr2	gfr2-Fw	AGGCTGTATTCCCAACATCC
	gfr2-Rv	ATGCCTTCTTCTTCTTCTCAG
nda2	nda2-Fw	CAAGCAGGTGTTCAAATCGG
	nda2-Rv	GCTCCAAACAGTACAACTCC
pma1	pma1-Fw	TCGAAAGATCTAGTCACCATC
	pma1-Rv	AGAAAAAGGTGGGAGGAAGG
tlh	tlh-Fw	GTGTGCTGTTACGAACAATAC
	tlh-Rv	TCGTTGTAGTGGTTGCTATAG

*This primer was added to all the RTase reaction for normalization.

Supplementary Table 4. Antibodies used in this study

Mouse monoclonal antibody	Clone	Source	Cat #	Reference
anti-CTD of RNA polymerase II	4H8	Novus Biologicals	NB200-598	
anti-di-methylated histone H3 (Lys-4)	MABI0303	MAB Institute, Inc.	MABI0303	
anti-tri-methylated histone H3 (Lys-4)	MABI0304	MAB Institute, Inc.	MABI0304	
anti-di-methylated histone H3 (Lys-9)	5.11	Lab stock		19
anti-alpha tubulin	DM1A	Santa Cruz Biotec.	sc-32293	

Rabbit monoclonal antibody	Clone	Source	Cat #
anti-acetylated histone H3 (Lys-56)	EPR996Y	abcam	ab76307

Rabbit polyclonal antibody	Source	Cat #
anti-acetyl-histone H3	Millipore	06-599
anti-acetyl-histone H4	Millipore	06-866
anti-C-terminal of histone H3*	Millipore	07-690
anti-C-terminal of histone H3**	abcam	ab1791
anti-C-terminal of histone H4	abcam	ab7311
anti-GFP	BioAcademia, Inc.	60-011

*This antibody was used for western blotting.
**This antibody was used for chromatin immunoprecipitation.

Supplementary Table 5. Gene sets for ChIP-seq analyses

Very high	High	Medium	Low
SPCC1739.13	SPBC16E9.05	SPAC21E11.06	SPAC977.17
SPBC32F12.11	SPAPB17E12.14c	SPBC29A10.07	SPBC2D10.13
SPAC4H3.10c	SPBC1711.08	SPAC1B1.01	SPCC1393.07c
SPAC9.09	SPCC1259.08	SPBC83.15	SPBC18E5.03c
SPBC1709.05	SPAC328.03	SPAC9G1.08c	SPAC15A10.10
SPBC215.05	SPBC17D11.05	SPBC8D2.11	SPAC458.06
SPBC1E8.05	SPBC18H10.02	SPBC2G2.14	SPCC1919.01
SPAC6B12.15	SPBC83.01	SPBC3H7.09	SPAC2F7.17
SPBC3D6.02	SPCC1281.01	SPBC16D10.03	SPAC323.03c
SPAC16.05c	SPBC16H5.08c	SPBC19G7.17	SPBC947.06c
SPAC25B8.12c	SPBP4G3.02	SPAC23D3.08	SPBC1773.13
SPAC1006.07	SPAC17G6.13	SPBP23A10.05	SPCC1183.05c
SPAC11E3.13c	SPCC306.11	SPCC1223.01	SPCC1682.06
SPBC1685.13	SPBC776.09	SPAC26H5.12	SPBC16C6.10
SPAC29E6.08	SPAC25G10.08	SPBC2G2.16	SPBC16G5.17
SPBC8E4.01c	SPAC23D3.12	SPBC3B8.01c	SPAC1002.19
SPAC29A4.02c	SPAC6G9.08	SPAC17G6.09	SPCC23B6.03c
SPAC9E9.03	SPBC365.16	SPAC57A10.02	SPBC1289.08
SPBC36.03c	SPAC17C9.13c	SPAC1527.03	SPBC21.07c
SPAC17D4.01	SPAC1071.09c	SPCC1235.02	SPAP27G11.14c
SPAC343.12	SPBC19C7.06	SPCC1682.04	SPAC1952.01
SPBC16A3.08c	SPBC56F2.08c	SPBC26H8.12	SPACUNK4.13c
SPBC2G5.05	SPAC29A4.04c	SPAC22F8.11	SPAPB1A10.02
SPBC2F12.14c	SPAC227.18	SPAC22E12.07	SPAC688.06c
SPBC1105.05	SPBC28F2.12	SPBC776.14	SPAC19B12.08
SPBC4B4.08	SPBC17G9.09	SPAC29B12.01	SPAC328.08c
SPCC18.14c	SPAC14C4.09	SPBC19C7.03	SPBC577.07
SPAC630.08c	SPAC26A3.01	SPCC1620.14c	SPCC11E10.03
SPAC1805.10	SPAC1B3.05	SPAC2C4.07c	SPBC27B12.05
SPCC1322.04	SPAC26A3.05	SPAC20H4.01	SPBC359.02
SPBC23E6.09	SPAC11D3.05	SPAC24C9.03	SPBC1271.01c
SPAC1F7.05	SPAC19G12.08	SPBC36B7.04	SPBC1711.11
SPAC140.02	SPAC3H1.11	SPBC2D10.18	SPBC1271.08c
SPBC660.16	SPBC1685.01	SPAC589.09	SPBC13E7.05
SPAC2F3.09	SPBP35G2.14	SPBC17D11.06	SPAC23H4.14
SPAC56F8.16	SPBC11C11.08	SPAC19B12.01	SPBC16H5.09c
SPAC1786.02	SPAPB2B4.01c	SPBC713.03	SPCC1442.02
SPACUNK4.16c	SPAC56E4.03	SPAC3H8.06	SPCC1620.03
SPBC660.11	SPAC24B11.13	SPBC21C3.11	SPCC1393.05
SPAC3A11.07	SPAC11G7.01	SPAC6F6.08c	SPCC1739.15
SPCC1393.08	SPAP14E8.02	SPAC1805.06c	SPAC977.16c
SPAPB1E7.07	SPBC18E5.07	SPBC1105.10	SPAC869.05c
SPBC409.08	SPAC26F1.13c	SPAC30D11.14c	SPBC16E9.07
SPBC8E4.02c	SPAC19E9.03	SPAC30D11.06c	SPAC1687.20c
SPAC821.09	SPAC6C3.04	SPBC6B1.02	SPCC162.04c
SPBC16E9.13	SPCC18.01c	SPBC23E6.08	SPAC212.04c
SPBC32F12.10	SPBC14C8.14c	SPAC4A8.07c	SPAPJ691.02
SPBC1683.01	SPCC1223.08c	SPBC19F8.05	SPAC1486.08
SPBC3H7.02	SPBC336.03	SPBC1604.02c	SPCC1906.04
SPBC428.11	SPBC713.12	SPAC23D3.06c	SPCC1884.02

Supplementary Table 6. Results of read mapping onto the reference genome

Strain	Analyzed reads	Read length (base)	Reference	Mapped reads	Mean depth	Relative depth to chromosome I
HKM-35	32,740,936	75	mitochondria	2,571,475	9925	61.58
			chromosome I	11,988,899	161.2	1
			chromosome II	9,723,023	160.6	0.9967
			chromosome III	7,472,039	228.5	1.418
			total	31,755,436	189.2	
HKM-1269	31,802,910	71	mitochondria	2,381,308	8701	57.16
			chromosome I	11,960,902	152.2	1
			chromosome II	9,938,403	155.4	1.021
			chromosome III	7,239,463	209.6	1.377
			total	31,520,076	177.7	

Supplementary Table 7. Mutation analysis of *de novo* assembled contigs

	HKM-35	HKM-1269
Number of contigs	5896	4155
Total length of contigs (bp)	11603987	12326252
Mean length of contigs (bp)	1968	2967
Median length of contigs (bp)	145	54
Number of mapped contigs (bp)	3080	1325
Mean length of mapped contigs (bp)	3705	9165
Median length of mapped contigs (bp)	2256	1577
Total length of mapped regions (bp)	11410153	12143401
% mapped	90.6	96.4
Total length of unmapped regions (bp)	1181098	447850
% unmapped	9.38	3.51
Contigs containging the *spt6* locus		
Contig name	NODE_934	NODE_772
Length (bp)	16,830	37,362
DDBJ ID	AB762286	AB762285

3. Supplementary Methods:

Genetic manipulations. All strains used were derived from the wild-type strains 968, 972, and 975. The fission yeast strains and primers for genetic manipulations used in this study are listed in Supplementary Tables 1 and 2, respectively. Unless stated otherwise, yeast extract (YES) and Edinburgh minimal (EMMS) media were supplemented with 250 mg/L each of adenine, leucine, and uracil. G418 sulfate (Calbiochem, 345810) and hygromycin B (Nacalai, 07296-24) were added to YES at concentrations of 100 and 200 mg/L, respectively, to select for antibiotic-resistant clones. 5-Fluoroorotic acid (5-FOA) (Wako, 064-03664), a counter-selective drug for *ura4*$^{+}$ expression, was added to YES at 0.1%. Deletion and tagging of chromosomal genes were performed with the pFA6a- and pCR2.1-based plasmid modules[38-40] and gene-specific primers. Error-prone PCR of *spt6*$^{+}$ was used to generate *spt6-M1242**. Coding sequences and epitopes of newly generated strains were sequenced to confirm that no additional mutations were introduced. Standard genetic crossing was performed to generate derivative strains.

To obtain strains expressing EGFP-fused Swi6^{HP1}, the *swi6*$^{+}$-coding region (-1214~+1290) was first cloned into pBluescript with an *ura4*$^{+}$ marker gene. A *Bam*HI restriction site was introduced immediately after the ATG codon using site-directed mutagenesis, and the DNA fragment encoding EGFP was inserted into this *Bam*HI site. The resulting plasmid was cleaved with *Mfe*I and introduced into the original *swi6*$^{+}$ locus. To replace the wild-type *swi6*$^{+}$ allele with the *egfp-swi6*$^{+}$ allele, strains that had lost the *ura4*$^{+}$ gene through internal homologous recombination were isolated using a counter-selective medium containing 5-FOA.

Isolation of silencing-defective mutants. In order to find novel heterochromatin regulators that work in conjunction with RNA polymerase II[19,41], a forward genetic screen for mutants that induce derepression of marker genes inserted in the pericentromere regions was performed. The wild-type strain used was HKM-35, which is genetically identical to HKV-89[19]. Strain HKM-35 is a derivative of FY2002[21], which possesses *ade6*$^{+}$ and *ura4*$^{+}$ marker genes in the pericentromeric *otr1R* and *imr1L* repeats, respectively. Silencing of the marker genes is derepressed by deletion of Clr4^{Suv39h} and Dcr1^{Dicer}, a homolog of Dicer[23,42]. The native *ade6* gene on chromosome III had been disrupted with *kanMX* to distinguish pseudo-positive Ade^{+} clones[19]. Cells growing on YES plates were exposed to 254-nm ultraviolet light at 180 J/m^{2} using a FUNA UV Crosslinker FS-800 (Funakoshi, Japan). Under these conditions, cell division stalled in almost all the cells, and half of the cells failed to form viable colonies. After nonselective overnight incubation in

liquid YES medium, cells were washed three times with PBS and spread on EMMS lacking adenine. After incubation for five days at 30˚C, Ade$^+$ colonies were picked and streaked on YES plates containing G418 to confirm that *ade6Δ::kanMX* had not been replaced with pericentromeric *ade6*$^+$. A total of 2001 out of 2386 Ade$^+$ clones were G418-resistant. In this kind of positive screen, transient Ade$^+$ "epi-clones" usually emerge, which gradually reestablish the silencing state. After four cycles of replica plating on YES medium containing a limited amount of adenine, only 20 clones were eventually selected as epigenetically stable "genetic clones," as they did not form red colonies during continuous passage. Sensitivity to 5-FOA in these 20 clones indicated that the *ura4*$^+$ marker gene was also derepressed.

Strains that harbor the *ade6-DS/E* allele in the *ade6* locus were used in a series of backcrossings. In contrast to harboring of the *ade6-m210* allele, which leads to formation of brighter colonies on YES plates with low adenine, strains that harbor the *ade6-DS/E* allele form darker colonies[21]. This enhanced pigmentation helped us to determine the degree of silencing defects. A total of 8 of the 20 mutants formed dark pink colonies on YES plates with low adenine, as did the *clr4Δ*^{Suv39h} and *dcr1Δ*^{Dicer} strains, suggesting the corresponding genes were in the known pathway[43,44]. In contrast, the remaining 12 mutants formed much brighter pink or white colonies that resembled the RNAi-defective RNA polymerase II mutant, *rpb2-m203*[19]. Of the 12 mutants, 6 were genetically separated from centromere 1 and segregated in a Mendelian manner, indicating that mutations somewhere in the chromosome arm were responsible for the phenotype. These candidates were backcrossed at least three times with wild-type strains. In this paper, we focused on a mutant designated as *K20*; which stands for the twentieth stable Ade$^+$ clone in the mutant pool designated as "K." Analyses of the other five mutants will be described elsewhere.

Identification of the corresponding mutation. Genomic DNA of the wild-type HKM-35 strain and the originally isolated *K20* strain HKM-1269, which did not undergo a genetic cross after the UV treatment, was extracted using a GIAGEN Genomic DNA Buffer Set (Catalog No. 19060) and Genomic-tip 500/G (Catalog No. 10262), according to the manufacturer's instructions. Whole genomic DNA was sequenced using Illumina GAIIx at the Dragon Genomics Center, TaKaRa Bio Inc. (Japan). Both 75-base (HKM-35) and 71-base (HKM-1269) paired-end reads were mapped to the reference genome (NC_001326.1, NC_003421.2, NC_003423.3 and NC_003424.3) and subjected to SNP and indel calling using BWA[45] and SAMtools[46]. The mean depth was over 150 for each strain (Supplementary Table 6). We found five unique transitions and one unique

transversion that turned out not to be genetically linked to the *K20* phenotype in the HKM-1269 genome. In a sophisticated analysis of read-depth though the genome, we noticed that there was a 231-bp region where the depth was less than 3, despite the high mean depth (Supplementary Fig. 1a). Analyses of *de novo* assembled contigs with Velvet[47] and MUMmer[48] consistently revealed a 246-bp deletion in a 37,362-bp contig (Supplementary Table 7 and Supplementary Fig. 1b). This 246-bp deletion was confirmed by Sanger sequencing (Supplementary Fig. 1c). We speculate that this deletion was the consequence of an unwanted recombination event between the two 9-bp elements (TCATCAACA) flanking the inner 237-bp sequence (Supplementary Fig. 1c). All of the HKM-1269 progenies with the *K20* phenotype possessed this deletion (n = 19, Supplementary Fig. 1d). This genetic linkage strongly suggested that the deletion was responsible for the phenotype. Indeed, reintroduction of this deletion into the wild-type genome resulted in the same silencing defect as in the original *K20* mutant (Supplementary Figs. 1e, f); therefore, we concluded that the 246-bp deletion was responsible for the *K20* phenotype. As this deletion hit the *spt6* gene, we named this allele *spt6-K20*.

Characterization of *spt6-K20*. Since integration of the *ade6*$^+$ and *ura4*$^+$ marker genes into pericentromeric repeats in the wild-type background results in epigenetic silencing[21], wild-type cells failed to grow on plates lacking adenine (EMMS -Ade), accumulated red pigment on plates containing a limited amount of adenine (YES low-Ade), and exhibited resistance to 5-FOA (YES 5-FOA) (Supplementary Fig. 2a). In contrast to the case with wild-type cells, *spt6-K20* cells were prototrophic for adenine, formed bright pink colonies and were sensitive to 5-FOA (Supplementary Fig. 2a), suggesting that the marker genes had been derepressed. Strand-specific quantitative RT-PCR (RT-qPCR) analyses confirmed increased expression of the *ade6*$^+$ marker gene as well as heterochromatic bidirectional transcripts from the pericentromeric *dg* and *dh* repeats and the subtelomeric *tlh* gene in *spt6-K20* cells (Supplementary Fig. 2b). We noticed that the expression levels of transcripts from the pericentromere detected by RT-qPCR analyses of *spt6* mutant cells were higher than those in *clr4*Δ$^{\mathrm{Suv39h}}$ and *dcr1*Δ^{Dicer} cells, which are defective in Lys-9 methylation and RNAi, respectively[20,23,49]. The *spt6* alleles *spt6-M1242**, which lacks a functional tSH2 domain, and the complete deletant (*spt6*Δ), caused similar silencing defects (Supplementary Figs. 2a, b). Both *spt6-M1242** and *spt6*Δ cells grew much slower than *spt6-K20* cells (Supplementary Fig. 2a), indicating that Spt6 plays an important role in cell growth. In order to manipulate the Spt6 protein, we fused GFP (S65T) to the C-terminus of Spt6. The GFP did not interfere with the silencing function of Spt6

(Supplementary Fig. 2c). ChIP-qPCR analysis demonstrated that Spt6-GFP localizes primarily in the euchromatic *act1* gene, and to a lesser extent in the heterochromatic regions (Supplementary Fig. 2d). We noticed that the nuclear fluorescence intensity of Spt6-GFP containing the *K20* deletion was significantly lower than that of the wild-type version (Supplementary Fig. 2e). Western blotting analysis also demonstrated a reduction in the level of Spt6-GFP due to the *K20* deletion (Supplementary Fig. 2f). Because of the reduced protein level, occupancy of Spt6-GFP in the *act1* gene was significantly reduced (Supplementary Fig. 2g). Microarray-based transcriptome analysis showed that in general the same genes exhibited a significant change in expression level in both *spt6-K20* and *spt6*Δ cells (Supplementary Fig. 2h). These results indicate that *spt6-K20* is a hypomorphic allele that causes a significant decrease in expression of Spt6 protein.

Microscopic observation. Living cells were mounted in YES medium containing 0.5% low-melting-point agarose (01161-12, Nacalai) on a glass slide preheated to 37˚C. The GFPs were observed under an FV1000-D confocal microscope (Olympus, Japan) equipped with an UPlanSApo 100X/1.40 objective lens, as per the manufacturer's instructions. Acquisition parameters were fixed through experiments comparing the brightness of fluorescence.

RNA preparation, RT-qPCR, northern blotting, and transcriptome analysis. Total RNA was extracted from exponentially growing cells as described elsewhere[19]. Reverse transcription was performed using ReverTraAce (Toyobo, TRT-101), according to the manufacturer's instructions. The primers used in RT-qPCR analyses are listed in Supplementary Table 3. In addition to strand-specific primers for the target genes, a primer complementary to the 28S rRNA was added to the reverse transcription reaction for normalization. The concentration of target sequences relative to the concentration of 28S rRNA was determined using SYBR Premix *ExTaq* (TaKaRa, RR041A) and a Thermal Cycler Dice Real Time System TP800 (TaKaRa), according to the manufacturer's instructions. Northern blotting analysis of siRNA was performed as described elsewhere[50].

For microarray-based transcriptome analysis, a custom-made array with sense and antisense probes for 4981 unique genes contained in the Ensembl database (http://fungi.ensembl.org/, release-9) was used. Library construction, hybridization and intensity calling were performed at Filgen, Inc. (http://www.filgen.jp). Poly-A RNA was labeled using an Amino Allyl MessageAmp™ II aRNA Amplification Kit (Life Technologies Corporation, AM1753) and a Cy5 Mono-Reactive Dye Pack (GE Healthcare Bio-Science Corp., PA25001). Labeled aRNA (4.0 μg) in 40 mM Tris-acetate (pH 8.1), 0.1 M potassium

acetate, and 30 mM magnesium acetate was denatured at 94˚C, cooled on ice, mixed with 10 µg of poly dA and 10 µg of budding yeast tRNA, and ethanol precipitated. Labeled nucleic acid was resuspended in hybridization buffer (5X SSC, 0.1% SDS, 10% formamide), incubated at 70˚C for 3 min, and applied to the microarray. Microarrays were incubated at 42˚C for 20 hours, washed with 2X SSC containing 0.1% SDS for 10 min at 42˚C, washed with 0.1X SSC containing 0.1% SDS for 10 min at 25˚C, washed twice with 0.1X SSC for 5 min at 25˚C, and then washed once with 0.1X SSC for 1 min at 25˚C. Microarrays were scanned using GenePix 4000B (Molecular Devices). Array-Pro Analyzer® Ver.4.5 (Media Cybernetics, Inc.) was used to determine the signal intensity of each spot and its local background. Raw intensity data were quantile normalized and analyzed using Microarray Data Analysis Tool Ver. 3.0 software (Filgen, Inc.). Genes that showed a significant change in expression level (>1.5-fold, $P<0.05$, Student's t-test) were chosen for subsequent analysis. Fisher's exact test was carried out in the R-statistical environment (http://www.R-project.org). Venn diagrams were drawn using the VennDiagram package[51].

Chromatin immunoprecipitation. Chromatin immunoprecipitation was performed as described elsewhere[19], with some modifications as described below. For immunoprecipitation of K9me2 and EGFP-Swi6^{HP1}, cells were incubated for an additional 1 h at 18˚C. After fixation with 1% formaldehyde and quenching with 125 mM glycine, cells were disrupted by shaking with glass beads (11079-105, BioSpec) in a Multi-Beads Shocker (MB400U, Yasui Kikai, Japan). A Bioruptor (UCD-250, COSMO BIO, Japan) was used for DNA shearing. The antibodies used in chromatin immunoprecipitation procedures are listed in Supplementary Table 4. A QIAquick PCR Purification Kit (28106, QIAGEN) was used to purify immunoprecipitated DNA. The relative concentration of target sequences was determined using the primers listed in Supplementary Table 3 as described in the previous section for RT-qPCR. Fold enrichment was calculated by comparing the relative quantity of targets in ChIP samples to those in the input samples, using one of the following three internal control regions for normalization; 1) *act1* (*SPBC32H8.12c*), a constitutively transcribed euchromatic gene on chromosome II, in which K9me2 is absent, 2) the gene-free region (*gfr2*) between *wis1* and *SPBC409.08*[52], where Pol II does not transcribe, and 3) *atp6* (*SPMIT.07*), a gene in the mitochondrial genome, to which nuclear proteins are not attached.

High-throughput sequencing of ChIP and input DNA using an Illumina HiSeq 2000 sequencer was done at BGI (http://www.genomics.cn/en/). Approximately 10-million single-end 49-base reads were mapped onto the 972 (mating-type: *h*$^-$) reference genome

and processed using BWA[45] and SAMtools[46]. The mating-types of tested strains were *h*⁻. Therefore, these strains do not have the *K*-region in the mating-type locus that is partly homologous to the pericentromeric repeats[53]. Random mapping of repeat-related reads was allowed in the study of protein occupancy in heterochromatic regions. Regions with significant change (P<0.001) in protein occupancy were identified by MACS[37,54]. Wiggle format pileup files (step size: 10 bp) generated by MACS were processed in the R-statistical environment. We built an R package that imports the MACS pileup files to normalize them and extract tag counts with respect to gene annotations. Quantile normalization[55] was applied genome-wide to compare protein occupancy to avoid major biases in sample preparation. The dynamic range of histone H3 occupancy in chromosome arms was not as high as that of Pol II (Supplementary Figs. 7b, c). In addition, the histone H3 occupancy tends to be relatively high in the repeat elements. As a consequence, some local biased peaks were generated in the pericentromeric repeats by the quantile normalization. Therefore, to analyze histone H3 occupancy in the pericentromere, linear normalization[56] was applied to avoid such biases. Colored images for the pericentromeric region in Figure 1d were generated by IGV[57]. In the GFF annotation file obtained from Pombase (ftp://ftp.sanger.ac.uk; pombe_09052011.gff), 4,376 genes had unique 5'- and 3'-UTRs. The 5'-ends of 5'-UTRs and the 3'-ends of 3'-UTRs were considered to be transcription start sites (TSS) and transcription termination sites (TTS), respectively. A total of 2,780 long transcript length genes (>1.5 kb) were selected. These genes were sorted according to the FPKM (fragments per kilobase of transcribed region per million mapped reads) values of Pol II to obtain gene sets of "Very high," "High," "Medium," and "Low" expression levels; Each gene set contained 50 genes (Supplementary Figs. 7a-c). Systematic gene names in the gene sets are listed in Supplementary Table 5. The Wilcoxon signed rank test was applied for assessing the differences of tag counts at each 10-bp interval between strains.

Micrococcal nuclease treatment followed by quantitative PCR analysis. Micrococcal nuclease (MNase) digestion of genomic DNA was performed as described elsewhere[58], with some modifications. The cell walls of growing cells fixed with 1% formaldehyde were digested with 0.5 mg/mL of Zymolyase 20T in sorbitol/Tris buffer. Spheroplasts were treated for 20 min at 37˚C with 10 U/mL of MNase (TaKaRa, 2910A) in NP buffer[58] containing 1 mM 2-mercaptoethanol and 0.5 mM spermidine. After treatment with RNase A and proteinase K, residual DNA in the supernatant was purified using a QIAquick PCR purification kit. The relative concentration of target sequences was determined as

described above for RT-PCR with primers listed in Supplementary Table 3. For normalization purposes, the resistance of target sequences to MNase was calculated by comparing the relative quantity of the target sequences in MNase-treated and untreated samples using the mitochondrial *atp6* gene, which is nucleosome-free and therefore most sensitive to MNase, as a reference.

Western blotting analysis of histones. Unfixed cells growing exponentially in YES medium were collected and disrupted by shaking with beads as described above for ChIP analyses. The extraction buffer[19] contained Triton X-100. After centrifugation of the extract, the pellet containing insoluble material was resuspended in the extraction buffer to obtain the chromatin fraction. Samples were separated by SDS-PAGE and histones were detected using the antibodies listed in Supplementary Table 4.

4. Supplementary references:

38 Bahler, J. *et al.* Heterologous modules for efficient and versatile PCR-based gene targeting in Schizosaccharomyces pombe. *Yeast* **14**, 943-951 (1998).

39 Krawchuk, M. D. & Wahls, W. P. High-efficiency gene targeting in Schizosaccharomyces pombe using a modular, PCR-based approach with long tracts of flanking homology. *Yeast* **15**, 1419-1427 (1999).

40 Sato, M., Dhut, S. & Toda, T. New drug-resistant cassettes for gene disruption and epitope tagging in Schizosaccharomyces pombe. *Yeast* **22**, 583-591 (2005).

41 Djupedal, I. *et al.* RNA Pol II subunit Rpb7 promotes centromeric transcription and RNAi-directed chromatin silencing. *Genes Dev.* **19**, 2301-2306 (2005).

42 Ekwall, K. *et al.* Mutations in the fission yeast silencing factors clr4+ and rik1+ disrupt the localisation of the chromo domain protein Swi6p and impair centromere function. *J. Cell Sci.* **109**, 2637-2648 (1996).

43 Goto, D. B. & Nakayama, J. I. RNA and epigenetic silencing: Insight from fission yeast. *Develop. Growth Differ.* **54**, 129-141 (2012).

44 Reyes-Turcu, F. E. & Grewal, S. I. Different means, same end-heterochromatin formation by RNAi and RNAi-independent RNA processing factors in fission yeast. *Curr. Opin. Genet. Dev.* **22**, 156-163 (2012).

45 Li, H. & Durbin, R. Fast and accurate short read alignment with Burrows-Wheeler transform. *Bioinformatics* **25**, 1754-1760 (2009).

46 Li, H. *et al.* The Sequence Alignment/Map format and SAMtools. *Bioinformatics* **25**, 2078-2079 (2009).

47 Zerbino, D. R. & Birney, E. Velvet: algorithms for de novo short read assembly using de Bruijn graphs. *Genome Res.* **18**, 821-829 (2008).

48 Kurtz, S. *et al.* Versatile and open software for comparing large genomes. *Genome Biol.* **5**, R12 (2004).

49 Bannister, A. J. *et al.* Selective recognition of methylated lysine 9 on histone H3 by the HP1 chromo domain. *Nature* **410**, 120-124 (2001).

50 Iida, T., Kawaguchi, R. & Nakayama, J. Conserved ribonuclease, Eri1, negatively regulates heterochromatin assembly in fission yeast. *Curr. Biol.* **16**, 1459-1464 (2006).

51 Chen, H. & Boutros, P. C. VennDiagram: a package for the generation of highly-customizable Venn and Euler diagrams in R. *BMC Bioinformatics* **12**, 35 (2011).

52 Choi, E. S., Shin, J. A., Kim, H. S. & Jang, Y. K. Dynamic regulation of replication independent deposition of histone H3 in fission yeast. *Nucleic Acids Res.* **33**, 7102-7110 (2005).

53 Grewal, S. I. & Klar, A. J. A recombinationally repressed region between mat2 and mat3 loci shares homology to centromeric repeats and regulates directionality of mating-type switching in fission yeast. *Genetics* **146**, 1221-1238 (1997).

54 Zhang, Y. *et al.* Model-based analysis of ChIP-Seq (MACS). *Genome Biol.* **9**, R137 (2008).

55 Bolstad, B. M., Irizarry, R. A., Astrand, M. & Speed, T. P. A comparison of normalization methods for high density oligonucleotide array data based on variance and bias. *Bioinformatics* **19**, 185-193 (2003).

56 Zaratiegui, M. *et al.* RNAi promotes heterochromatic silencing through replication-coupled release of RNA Pol II. *Nature* **479**, 135-138 (2011).

57 Thorvaldsdottir, H., Robinson, J. T. & Mesirov, J. P. Integrative Genomics Viewer (IGV): high-performance genomics data visualization and exploration. *Brief. Bioinform.* **14**, 178-192 (2012).

58 Lantermann, A. *et al.* Genome-wide mapping of nucleosome positions in Schizosaccharomyces pombe. *Methods* **48**, 218-225 (2009).
